# Editorial: The Impact of Ultra-Processed Food Consumption on Health in Low- and Middle-Income Countries

**DOI:** 10.3389/ijph.2025.1609418

**Published:** 2026-01-05

**Authors:** Marialaura Bonaccio, Sukshma Sharma, Licia Iacoviello

**Affiliations:** 1 Research Unit of Epidemiology and Prevention, Mediterranean Neurological Institute Neuromed (IRCCS), Pozzilli, Italy; 2 Department of Medicine and Surgery, Universita LUM Giuseppe Degennaro, Casamassima, Italy

**Keywords:** epidemiology, food processing, health, low-and middle-income countries (LMICs), ultra-processed food (UPF)

## Introduction

In recent decades food processing has drastically changed to address consumer preferences and has led to higher demand for food items with longer shelf-life and improved palatability ultimately achieving this by adding natural or artificial ingredients which may impact on the nutritional quality of these foods, which are often characterized by high fat, sugar and salt contents [1]. Ultra-processed foods (UPFs) are defined as formulations made largely or entirely with cheap industrial sources of substances extracted from food, often chemically modified with additives and with a small amount of whole food using a series of processes [2]. Most importantly, UPFs are deliberately designed to be highly palatable and appealing, with extended shelf lives, and can be eaten conveniently in any setting, and their formulation, presentation and marketing often promote overconsumption [3]. Robust evidence from rigorously conducted cohort studies shows a clear link between UPF intake and adverse health outcomes, including reduced survival and increased risk of major chronic diseases [5], often independent of overall nutritional quality [6].

UPFs have progressively displaced traditional diets globally, and now constitute a major part of dietary intake, accounting for up to 50% of the energy intake in high-income countries [4]. Between 2007 and 2022, annual sales of UPFs (initially under 150 kg per person) increased across low-, lower-middle-, and upper-middle-income countries, as well as in all lower-income regions [5].

However, evidence on the impact of UPFs on populations within the low- and middle-income countries (LMICs) is limited and inconclusive, making it paramount to explore the implications of the rising UPF consumption within these countries.

This is particularly important given that LMICs account for the majority of the world’s population and are therefore at risk of a substantial public health burden. Importantly, exploration of UPFs in LMICs may bring to light the impact of common contributing factors including ethnicity, ingredients, tropical food consumption, occupational stress, and genetic or environmental factors.

## Key Highlights of the Special Issue

This Special Issue features seven papers that explore diverse topics in the field of UPF consumption and health outcomes within the LMICs settings, making the findings and insights all the more valuable to public health knowledge. The seven papers in this Special Issue explored the impact of UPF consumption in diverse conditions including, across different life stages, and various health outcomes making the findings widely interesting. They highlight that in Senegal, amongst adults, higher UPF consumption was linked to critical nutrients associated with risk of non-communicable diseases including higher total fat, free sugars and lower protein intakes (Kébé et al.); they compare and state that adults with type 2 diabetes mellitus had an overall lower UPF consumption than those without diabetes emphasizing the importance of promoting healthy eating habits to limit comorbidities such as diabetes mellitus (Mahajan et al.); they urged to examine the impact of higher UPF intake on adiposity and metabolic disturbances amongst adolescents (Ghosh and Muley); they find that most children and adolescents consumed unhealthy UPFs daily and had an overall unhealthier lifestyle in the Mediterranean region (Rosi et al.); they investigate that in slum settings in Kenya, adolescents with higher energy intakes from UPF had highest total energy, total fat, and saturated fat and lowest protein, fibre, and minerals such as iron, calcium and zinc intakes (Wanjohi et al.); they urge additional studies to examine the impact of UPF consumption on cognitive performance needs in adolescents within low-income settings (dos Santos et al.); and demonstrate that at a community level in South Brazil there was an increased consumption of traditional food appreciating local culture and lower UPF intake as a result of effective nutritional counselling intervention (Pacheco et al.).

Future research should prioritize comprehensive, longitudinal studies in LMICs to better understand the health impacts of UPF consumption across the life course. Particular attention should be given to the interplay of context-specific factors such as ethnicity, local ingredients, tropical diets, occupational stress, and genetic or environmental influences. Further investigations are also needed to evaluate effective interventions for reducing UPF intake and promoting traditional, nutrient-rich diets, particularly among children, adolescents, and vulnerable populations. Such evidence could inform culturally appropriate public health policies and nutrition strategies to mitigate the growing burden of diet-related chronic diseases in LMICs.

## References

[1] Martini D Godos J Bonaccio M Vitaglione P Grosso G . Ultra-Processed Foods and Nutritional Dietary Profile: A Meta-Analysis of Nationally Representative Samples. Nutrients (2021) 13(10):3390. 10.3390/nu13103390 34684391 PMC8538030

[2] Monteiro CA Cannon G Levy RB Moubarac JC Louzada ML Rauber F Ultra-Processed Foods: What They Are and How to Identify Them. Public Health Nutr (2019) 22:936–41. 10.1017/S1368980018003762 30744710 PMC10260459

[3] Touvier M Da Costa Louzada ML Mozaffarian D Baker P Juul F Srour B . Ultra-Processed Foods and Cardiometabolic Health: Public Health Policies to Reduce Consumption Cannot Wait. BMJ (2023) 383:e075294. 10.1136/BMJ-2023-075294 37813465 PMC10561017

[4] Elizabeth L Machado P Zinöcker M Baker P Lawrence M. Ultra-Processed Foods and Health Outcomes: A Narrative Review. Nutrients (2020) 12:1955. 10.3390/NU12071955 32630022 PMC7399967

[5] Monteiro CA Louzada ML Steele-Martinez E Cannon G Andrade GC Baker P Ultra-Processed Foods and Human Health: The Main Thesis and the Evidence. The Lancet (2025) 406:0–2684. 10.1016/S0140-6736(25)01565-X 41270766

[6] Bonaccio M Di Castelnuovo A Ruggiero E Costanzo S Grosso G De Curtis A Joint Association of Food Nutritional Profile by Nutri-Score Front-of-Pack Label and Ultra-Processed Food Intake With Mortality: Moli-Sani Prospective Cohort Study. BMJ (2022) 378:e070688. 10.1136/bmj-2022-070688 36450651 PMC9430377

